# Influence of Demographic Factors on Long-Term Trends of Premature Mortality and Burden Due to Liver Cancer: Findings From a Population-Based Study in Shanghai, China, 1973–2019

**DOI:** 10.3389/fpubh.2022.808917

**Published:** 2022-02-15

**Authors:** Zheng Luo, Yongbin Zou, Jiaxin Xie, Hui Cao, Yichen Chen, Yibo Ding, Xiaopan Li, Yang Deng, Lile Wu

**Affiliations:** ^1^Department of Neurology, Shanghai University of Medicine & Health Sciences Affiliated Zhoupu Hospital, Shanghai, China; ^2^Department of Oncology, Chenzhou First People's Hospital, Chenzhou, China; ^3^Department of High Altitude Operational Medicine, Army Medical University, Chongqing, China; ^4^Center for Disease Control and Prevention of Pudong New Area, Fudan University Pudong Institute of Preventive Medicine, Shanghai, China; ^5^Department of Epidemiology, Second Military Medical University, Shanghai, China; ^6^School of Public Health, Shandong First Medical University & Shandong Academy of Medical Sciences, Tai'an, China; ^7^Department of Hepatobiliary Surgery, The Affiliated Hospital of Southwest Medical University, Luzhou, China

**Keywords:** liver cancer, mortality, years of life lost, difference decomposition method, demographic factors

## Abstract

**Objective:**

Liver cancer is one of the most common causes of cancer-related death. Understanding how demographic factors influence mortality due to liver cancer is crucial for optimizing disease-control strategies. We aimed to characterize the long-term trends in the mortality and years of life lost (YLL) of liver cancer in Shanghai, China, 1973–2019, and quantitatively analyze the contributions of demographic and non-demographic factors on the mortality of liver cancer.

**Methods:**

Using mortality data from the Mortality Registration System of Pudong New Area, the largest district of Shanghai with a population of permanent resident of 5.68 million, during 1973–2019, we analyzed the temporal trends for the mortality rates and YLL by Joinpoint Regression Program. The difference decomposition method was employed to estimate the increasing mortality rates related to demographic and non-demographic factors.

**Results:**

A total of 21,530 deaths from liver cancer occurred from 1973 to 2019. The crude mortality rates (CMR) and age-standardized mortality rate by Segi's world standard population (ASMRW) of liver cancer were 26.73/10^5^ person-years and 15.72/10^5^ person-years, respectively. The CMR, ASMRW, and YLL rates of liver cancer showed significantly decreasing trends in males, females and the total population from 1973 to 2019, whereas the upward trends in the YLL were seen in males, females and the total population (all *P* < 0.05). A significant upward trend was observed in the increased CMR caused by demographic factors, but the changing rate caused by non-demographic factors decreased.

**Conclusions:**

The CMR and ASMRW of liver cancer continually decreased although YLL increased during 1973–2019 in Pudong New Area, Shanghai. The demographic factors, especially aging, might be responsible for the increase in the mortality of liver cancer. More effective prevention strategies tailored to liver cancer are needed to further reduce its disease burden in the elderly population.

## Introduction

Liver cancer is expected to rank as the sixth most common cancer and the third leading cause of cancer-related death, with an estimated 906,000 new cases and 830,000 deaths occurring worldwide in 2020 (1). China accounts for more than half of the world's incident and death cases of liver cancer. Liver cancer is the most commonly diagnosed cancer and the first leading cause of cancer-related death in men younger than 60 years old, and it remains a serious threat to human health and is the main cause of premature mortality (2, 3). In China, during 1990–2017, age-standardized incidence rate of liver cancer decreased from 27.16/10^5^ to 26.04/10^5^, and age-standardized mortality rate decreased from 26.72/10^5^ to 21.30/10^5^, respectively (4). In addition, the liver cancer-caused years of life lost (YLL) increased by 21.27% (from 644 per 100,000 population to 781 per 100,000 population) (5). It is shown that the incidence rate of liver cancer declines in China, but the burden increases. The incidence and mortality rates may not completely reflect the burden of liver cancer, because it harms the younger population more than others. YLL is an important measure quantifying the burden of cancer, and the calculation of YLL is based on the age at death and the number of deaths at each age, eliminating the mismatch of disease impact solely derived from the number of deaths (6).

The increasing burden of liver cancer is possibly attributed to demographic, epidemiological, and health transitions including explosive population growth, aging and unhealthy lifestyles (5). China has experienced rapid demographic and non-demographic transitions with fast-paced urbanization, aggravated aging, and environmental pollution in the past few decades (7). A wide range of demographic and non-demographic factors are associated with liver cancer, such as age, gender, race/ethnicity, chronic hepatitis B virus and/or hepatitis C virus infection, alcohol abuse, smoking, non-alcoholic fatty liver disease, metabolic syndrome, diabetes, obesity, aflatoxin B_1_, dietary habit, and genetic susceptibility (8, 9). Demographic factors are referred to age, gender, residence, education level, race/ethnicity, and family relationships. An age-period-cohort analysis showed that effect attributed to population aging was the most important demographic factor in the mortality of liver cancer compared with period and cohort effect, and the mortality risk increased gradually with age (10). Non-demographic factors such as economic level, access to healthcare services, improvement of medical technology, promotion of residents' health awareness, and changed living environment are caused by circumstances beyond the individual's control (11). However, the contribution of demographic and non-demographic factors on the mortality of liver cancer have not been quantitatively analyzed in China.

Shanghai is the largest metropolis in China, with a permanent resident population of 24.87 million in 2020. Moreover, Shanghai is one of the earliest industrial cities, and residents in Shanghai were exposed to pollution from heavy industry from 1949 to 1980s. Thereafter, the Shanghai government carried out pollution prevention and ecological environment protection activities, focusing on prevention and control of industrial pollution. In 1988, hepatitis A epidemic in Shanghai promoted the development of public health infrastructure and improved residents' health awareness. As a result of rapid economic growth for nearly 30 years since the initiation of economic reform in 1979, Shanghai has become an upper-middle income region since 2006 (12). Thus, Shanghai is the forerunner of demographic and socioeconomic development in China. Long-term epidemiologic studies are indispensable to characterize the influence of demographic and non-demographic factors on the trends of premature mortality. The cancer registration system in Shanghai was established in 1973, which was 14 years earlier than the establishment of Chinese national cancer registration system. The cancer registration data of Shanghai is of high quality and is approved by the World Health Organization (13). Pudong New Area is one of the largest cancer registries in China with a population of permanent resident of 5.68 million, accounting for approximately 23% of the total permanent resident population in Shanghai in 2020. In this population-based study, we aimed to analyze the long-term trends in the mortality and YLL due to premature mortality in people with liver cancer, and characterize the influence of demographic and non-demographic factors on the mortality using difference decomposition method, which may help in optimizing control strategies for liver cancer.

## Materials and Methods

### Data Source

The mortality data of registered permanent residents with liver cancer during 1973–2019 were derived from the Mortality Registration System of Pudong New Area, Shanghai. The mortality registration system covers medical institutions of all levels, and data are checked against local population registry monthly to ensure the accuracy (14). For deaths occurring outside the medical institutions, professionals from community sanitary service center collect the relevant data using verbal autopsy. Periodic evaluations, data cleaning and compilation are performed to ascertain the completeness of death data. The population data were provided by the Public Security Bureau of Pudong New Area, Shanghai.

Since the data covered a long-time span of 47-years, death data were divided into three parts based on the different coding methods. Historical records before 1975 which were recorded in paper form were digitized and recoded according to the International Classification of Diseases, 8th version (ICD-8). Records for 1975–2001 were coded according to the ICD-9. Data recorded before 1975 and during 1975–2001 were converted into ICD-10 as described previously (14). Since 2002, death from liver cancer (C22) was directly classified according to the ICD-10. All causes of death from liver cancer were coded by rigorously trained clinicians, and each record was further verified by professionals at the Center for Disease Control and Prevention (CDC). Proportion of morphological verification (MV%) and percentage of cases identified with death certification only (DCO%) suggested overall quality of data was satisfied.

### Statistical Analysis

The crude mortality rates (CMR) and age-standardized mortality rates by Segi's world standard population (ASMRW) of liver cancer were calculated by gender and period, and expressed as per 100,000 (/10^5^). Poisson approximation method was used to compare the CMR and ASMRW between genders (13). Age-specific mortality rates were calculated for the following age groups: 0–4 years, 5–14 years, 15–29 years, 30–44 years, 45–59 years, 60–69 years, 70–79 years, and ≥80 years. YLL was used to investigate the burden of liver cancer according to method and formula proposed in previous studies (11, 15). Temporal trends in the mortality, YLL, and YLL rate from 1973 to 2019 were examined using Joinpoint Regression Program (version 4.3.1.0, National Cancer Institute, MD, USA) and expressed as an average annual percentage change (AAPC) with corresponding 95% confidence interval (CI). The *Z* test was employed to analyze whether the annual percent change (APC) was statistically different from zero. The quantitative contribution of demographic and non-demographic factors on the change of mortality rate during 1973–2019 were evaluated by the difference decomposition method. The change of mortality rate within each period of 5 years from 1980 to 2019 were compared with the rate during 1973–1979 which was set as a basis (15, 16). Other statistical analyses were conducted using SPSS 21.0 (SPSS, Inc., Chicago, IL). *P*-value of < 0.05 were considered to indicate statistically significant differences.

## Results

### Basic Information and Burden of Death From Liver Cancer

A total of 21,530 deaths from liver cancer were reported during 1973–2019, with 14,873 men and 6,657 women. The average age at death from liver cancer was 64.73 ± 14.00 years old, and the median age at death was 65.11 years old. The CMR and ASMRW of liver cancer were 26.73/10^5^ person-years and 15.72/10^5^ person-years, respectively. The CMR and ASMRW were 37.40/10^5^ person-years and 23.53/10^5^ person-years in males, while the corresponding rates were 16.33/10^5^ person-years and 8.39/10^5^ person-years in females. The CMR and ASMRW were 1.29-fold and 1.80-fold higher in males than in females, respectively (both *P* < 0.05). The highest CMR and ASMRW occurred during the periods of 1995–1999 and 1985–1989, respectively (Table 1).

**Table 1:** Baseline characteristics of deaths and burden in different genders and periods of liver cancer during 1973–2019.

**Characteristic**	**Deaths**	**Age at death**	**Age at death**	**CMR**	**ASMRW**	**YLL**	**YLL rate**
	**(*n*, %)**	**(Mean ±SD)**	**(Median)**	**(/10^**5**^)**	**(/10^**5**^)**	**(years)**	**(/10^**5**^)**
Gender							
Male	14,873 (69.08)	62.42 ± 13.58	62.25	37.40	23.53	297,043.26	746.91
Female	6,657 (30.92)	70.16 ± 13.44	72.16	16.33	8.39	86,286.64	211.63
Period							
1973–1979	1,009 (4.69)	60.71 ± 13.19	62.08	23.43	26.10	15,891.83	368.98
1980–1984	814 (3.78)	61.31 ± 12.98	62.23	24.61	25.19	12,533.06	378.86
1985–1989	877 (4.07)	61.66 ± 13.63	62.11	28.52	26.50	13,377.92	434.98
1990–1994	1,336 (6.21)	61.26 ± 13.74	62.62	28.29	22.79	20,529.84	434.72
1995–1999	3,555 (16.51)	61.61 ± 13.79	62.90	31.98	23.72	54,126.73	486.95
2000–2004	3,554 (16.51)	62.49 ± 14.21	63.70	29.59	18.66	52,799.54	439.62
2005–2009	3,545 (16.47)	64.25 ± 14.06	63.75	27.02	14.29	58,823.50	448.31
2010–2014	3,515 (16.33)	66.85 ± 13.50	66.27	25.11	11.60	45,996.68	328.64
2015–2019	3,325 (15.44)	69.38 ± 12.95	68.65	22.33	9.03	40,007.28	268.74
Total	21,530 (100.00)	64.73 ± 14.00	65.11	26.73	15.72	383,329.90	475.93

*ASMRW, age-standardized mortality rate by Segi's world standard population; CMR, crude mortality rate; SD, standard deviation; YLL, years of life lost*.

During 1973–2019, the YLL due to premature death from liver cancer was 383,329.90 years, and the YLL rate was 475.93/10^5^. YLL and YLL rate in males (297,043.26 years and 746.91/10^5^) were significantly higher than those in females (86,286.64 years and 211.63/10^5^). In terms of study period, the highest YLL and YLL rate occurred during the periods of 2005–2009 and 1995–1999, respectively (Table 1).

### Age-Specific Mortality and Burden of Premature Death

The number of population aged 45–59 years (6,381 deaths) who died of liver cancer was the most among total deaths, followed by those aged 60–69 years (5,335 deaths) and 70–79 years (4,825 deaths). The CMR was at a low level before 30 years, and it increased rapidly from 45 years and peaked after 80 years. The top three in YLL were in the age groups of 45–59 years, 60–69 years, and 70–79 years, which were 146,729.54 years, 95,675.50 years, and 62,587.87 years, respectively. The YLL rate in the age group of 70–79 years was the highest (1,294.16/10^5^), followed by the age group of 60–69 years (1,101.96/10^5^) and ≥80 years (1,010.40/10^5^) (Table 2).

**Table 2:** Age-specific mortality and burden premature death from liver cancer during 1973–2019.

**Age group (years)**	**Deaths (n)**	**Proportion (%)**	**CMR (/10^**5**^)**	**YLL (years)**	**YLL rate (/10^**5**^)**
0–4	9	0.04	0.26	363.30	10.45
5–14	14	0.07	0.17	586.68	7.32
15–29	128	0.59	0.80	4,335.65	27.02
30–44	1,797	8.35	9.27	49,231.02	254.09
45–59	6,381	29.64	35.95	146,729.54	826.58
60–69	5,335	24.78	61.45	95,675.50	1,101.96
70–79	4,825	22.41	99.77	62,587.87	1,294.16
≥80	3,041	14.12	128.99	23,820.34	1,010.40
Total	21,530	100.00	26.73	383,329.90	475.93

*ASMRW, age-standardized mortality rate by Segi's world standard population; CMR, crude mortality rate; YLL, years of life lost*.

### Trends of Mortality and Burden of Liver Cancer

**Figure 1:**
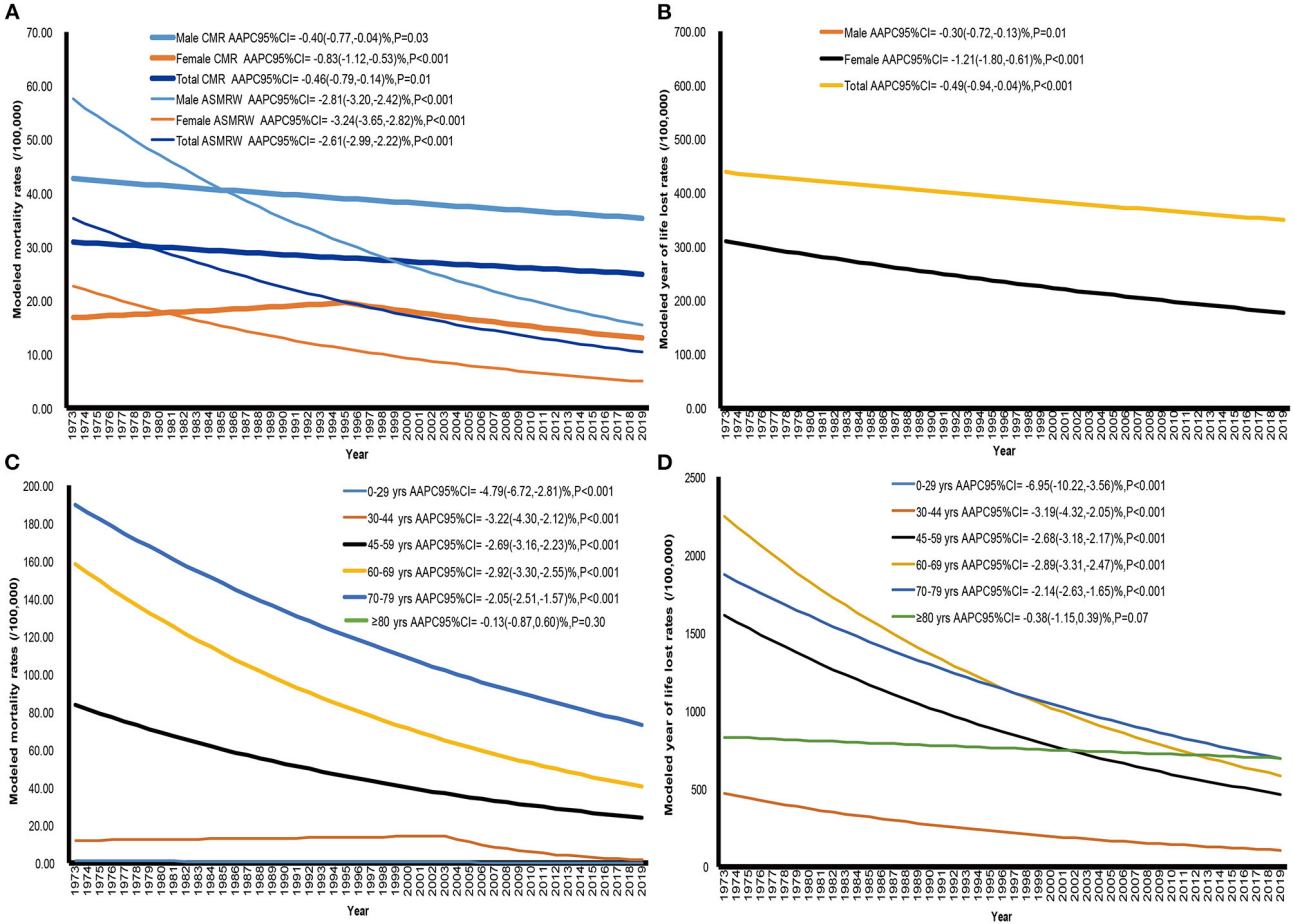
The trends of CMR, ASMRW, and YLL rate of persons with underlying cause of liver cancer death in genders and age groups in Pudong New Area, Shanghai, from 1973 to 2019. **(A)** CMR and ASMRW in genders, **(B)** YLL rate in genders, **(C)** CMR in age groups, **(D)** YLL rate in age groups. ASMRW, age-standardized mortality rate by Segi's world standard population (per 100,000); CMR, crude mortality rate (per 100,000); YLL, years of life lost (per 100,000).

The CMR, ASMRW, and YLL rates of liver cancer showed significantly decreasing trends in males, females and the total population from 1973 to 2019 (all *P* < 0.05) (Figures 1A, B and Table 3). However, upward trends in the YLL of liver cancer were seen in males, females and the total population (Table 3). In terms of age-specific mortality and burden, there were significant decreasing trends in the CMR and YLL rates among all age groups (all *P* < 0.001) except for the group of ≥80 years (Figures 1C, D and Table 3). The YLL decreased by 5.46% (95% *CI* = −9.29% to −1.48%, *P* = 0.01) per year in the age group of 0–29 years during the study period, while the YLL increased in the other age groups (all *P* < 0.05) (Table 3).

**Table 3:** Trends of CMR, ASMRW, YLL, YLL rate of liver cancer in genders and age groups during 1973–2019.

**Characteristic**	**Trend 1**		**Trend 2**		**Trend 3**		**Trend 4**		**Total (1973–2019) AAPC (95% *CI*)**
	**Period**	**APC** **(95% *CI*)**	**Period**	**APC** **(95% *CI*)**	**Period**	**APC** **(95% *CI*)**	**Period**	**APC** **(95% *CI*)**	
**CMR**									
Gender									
Male	1973–1998	1.72 (1.20, 2.24)^**^	1998–2019	−1.88 (−2.26, −1.49)^**^	–	–	–	–	−0.40 (−0.77, −0.04)^*^
Female	1973–1995	0.70 (−0.16, 1.57)	1995–2019	−1.69 (−2.12, −1.26)^**^	–	–	–	–	−0.83 (−1.12, −0.53)^**^
Total	1973–1997	1.57 (1.09, 2.05)^**^	1997–2019	−1.77 (−2.09, −1.45)^**^	–	–	–	–	−0.46 (−0.79, −0.14)^*^
Age groups (years)									
0–29	1973–2009	−1.86 (−4.51, 0.87)	2009–2019	−22.65 (−35.90, −6.65)^*^	–	–	–	–	−4.79 (−6.72, −2.81)^**^
30–44	1973–2003	0.60 (−0.80, 2.02)	2003–2019	−12.24 (−15.34, −9.03)^**^	–	–	–	–	−3.22 (−4.30, −2.12)^**^
45–59	1973–1991	0.35 (−1.14, 1.86)	1991–2019	−4.24 (−4.98, −3.50)^**^	–	–	–	–	−2.69 (−3.16, −2.23)^**^
60–69	1973–1995	−0.83 (−1.60, −0.05)^*^	1995–2019	−4.73 (−5.38, −4.07)^**^	–	–	–	–	−2.92 (−3.30, −2.55)^**^
70–79	1973–1995	0.19 (−0.95, 1.35)	1995–2019	−3.97 (−4.94, −3.00)^**^	–	–	–	–	−2.05 (−2.51, −1.57)^**^
≥80	1973–1976	58.32 (14.38, 119.15)^*^	1976–2019	−0.86 (−1.41, −0.29)^**^	–	–	–	–	−0.13 (−0.87, 0.60)
**ASMRW**									
Gender									
Male	1973–1991	0.18 (−0.84, 1.21)	1991–2019	−4.34 (−4.84, −3.83)^**^	–	–	–	–	−2.81 (−3.20, −2.42)^**^
Female	1973–1990	−0.11 (−1.46, 1.25)	1990–2019	−4.63 (−5.21, −4.05)^**^	–	–	–	–	−3.24 (−3.65, −2.82)^**^
Total	1973–1975	15.21 (−11.35, 49.73)	1975–1997	−0.83 (−1.49, −0.17)^*^	1997–2019	−4.82 (−5.41, −4.23)^**^	–	–	−2.61 (−2.99, −2.22)^**^
**YLL**									
Gender									
Male	1973–1991	2.45 (1.50, 3.41)^**^	1991–1996	34.13 (22.39, 47.01)^**^	1996–2019	−1.61 (−2.24, −0.97)^**^	–	–	4.75 (3.87, 5.64)^**^
Female	1973–1992	0.47 (−1.67, 2.66)	1992–1995	53.04 (−26.07, 216.79)	1995–2019	−1.77 (−3.24, −0.26)^*^	–	–	3.31 (2.39, 4.23)^**^
Total	1973–1992	1.96 (0.86, 3.07)^**^	1992–1995	56.99 (8.97, 126.18)^*^	1995–2019	−1.48 (−2.23, −0.73)^**^	–	–	4.29 (3.42, 5.16)^**^
Age groups (years)									
0–29	1973–2010	1.03 (−3.84, 6.15)	2010–2019	−46.09 (−64.37, −18.43)^**^	–	–	–	–	−5.46 (−9.29, −1.48)^*^
30–44	1973–2001	10.07 (8.09, 12.10)^**^	2001–2019	−10.94 (−14.03, −7.73)^**^	–	–	–	–	2.37 (0.65, 4.10)^*^
45–59	1973–1992	1.37 (0.19, 2.57)^*^	1992–1995	42.76 (−3.97, 112.23)	1995–2005	6.32 (2.54, 10.24)^**^	2005–2019	−6.44 (−8.16, −4.68)^**^	4.33 (3.32, 5.35)^**^
60–69	1973–1992	1.09 (−0.07, 2.26)	1992–1995	55.24 (5.11, 129.26)^*^	1995–2008	−2.84 (−5.05, −0.57)^*^	2008–2019	3.97 (1.27, 6.74)^**^	4.13 (3.40, 4.87)^**^
70–79	1973–1992	1.99 (0.46, 3.53)^*^	1992–1995	60.93 (−3.19, 167.52)	1995–2019	−0.91 (−1.96, 0.14)			4.86 (3.94, 5.79)^**^
≥80	1973–1976	52.06 (4.59, 121.06)^*^	1976–1992	4.74 (1.48, 8.11)^*^	1992–1995	54.56 (−26.88, 226.68)	1995–2019	5.00 (3.37, 6.65)^**^	9.44 (8.50, 10.39)^**^
**YLL rate**									
Gender									
Male	1973–1999	1.77 (1.23, 2.32)^**^	1999–2019	−3.26 (−4.03, −2.49)^**^	–	–	–	–	−0.30 (−0.72, −0.13)^*^
Female	1973–2019	−1.21 (−1.80, −0.61)^**^			–	–	–	–	−1.21 (−1.80, −0.61)^**^
Total	1973–2005	0.91 (0.53, 1.29)^**^	2005–2019	−5.12 (−6.34, −3.87)^**^	–	–	–	–	−0.49 (−0.94, −0.04)^**^
Age groups (years)									
0–29	1973–2010	−1.79 (−6.03, 2.64)	2010–2019	−41.02 (−59.25, −14.64)^*^	–	–	–	–	−6.95 (−10.22, −3.56)^**^
30–44	1973–2005	0.32 (−0.96, 1.61)	2005–2019	−14.27 (−17.99, −10.38)^**^	–	–	–	–	−3.19 (−4.32, −2.05)^**^
45–59	1973–1998	−0.69 (−1.72, 0.35)	1998–2019	−5.19 (−6.46, −3.89)^**^	–	–	–	–	−2.68 (−3.18, −2.17)^**^
60–69	1973–1995	−0.72 (−1.70, 0.26)	1995–2019	−4.76 (−5.58, −3.93)^**^	–	–	–	–	−2.89 (−3.31, −2.47)^**^
70–79	1973–1997	−0.13 (−1.21, 0.95)	1997–2019	−4.37 (−5.54, −3.19)^**^	–	–	–	–	−2.14 (−2.63, −1.65)^**^
≥80	1973–1977	38.94 (9.63, 76.09)^*^	1977–2019	−1.24 (−1.91, −0.58)^**^	–	–	–	–	−0.38 (−1.15, 0.39)

*AAPC, average annual percentage change; APC, annual percent change; ASMRW, age-standardized mortality rate by Segi's world standard population; CI, confidence interval; CMR, crude mortality rate*.

* *P < 0.05*.

** *P < 0.001*.

### Quantitatively Contribution of Demographic and Non-demographic Factors on the Change of CMR

**Figure 2:**
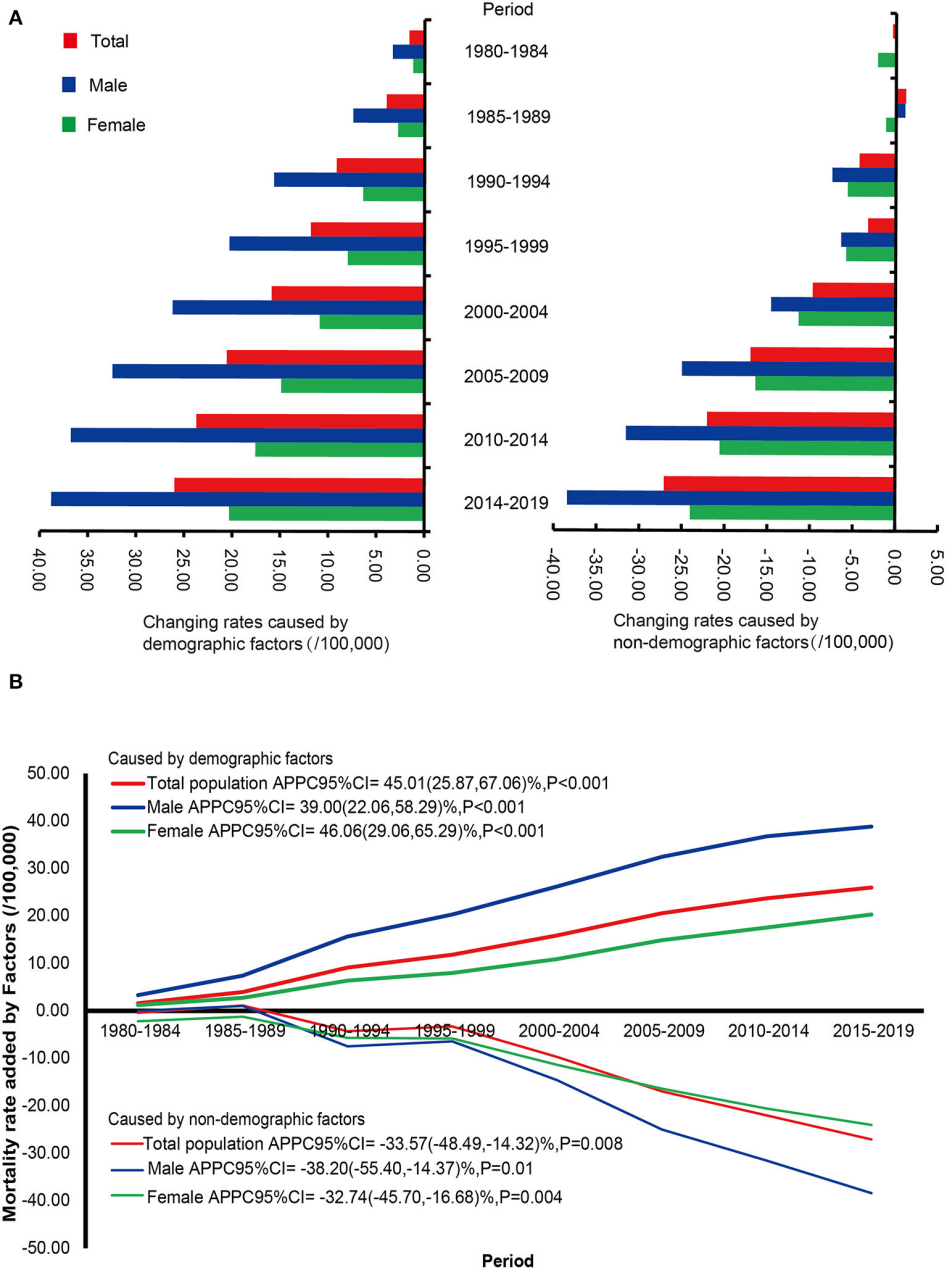
The increased rates caused by demographic and non-demographic factors and their proportion during the period from 1980 to 2019 compared with the crude mortality rate of liver cancer during 1973–1979 in Pudong New Area, Shanghai, China. **(A)** The increased rates caused by demographic and non-demographic factors, **(B)** The trends of changing values of crude mortality rate caused by demographic and non-demographic in genders. APPC, average period percentage change; CI, confidence interval.

The trends of changing values of CMR caused by demographic and non-demographic factors are shown in Figure 2 and Supplementary Table 1. Based on the CMR of liver cancer during 1973–1979, the changing value of mortality rates caused by demographic factors showed an increasing trend in males, with an average period percentage change (APPC) of 39.00% (95% *CI* = 22.06–58.29%, *P* < 0.001) from 1980 to 2019, while a significantly decreasing trend was observed in the changing value related to non-demographic factors [APPC (95% *CI*) = −38.20% (−55.40 to −14.37%), *P* = 0.01]. In females, the change of CMR related to demographic factors increased by 46.06% (95% *CI* = 29.06–65.29%, *P* < 0.001) during 1980–2019, but the change related to non-demographic factors decreased with an APPC of −32.74% (95% *CI* = −45.70 to −16.68%, *P* = 0.004). In the total population, a significant upward trend was observed in the increased CMR caused by demographic factors during 1980–2019 [APPC (95% *CI*) = 45.01% (25.87–67.06%), *P* < 0.001], whereas the changing rate caused by non-demographic factors decreased [APPC (95% *CI*) = −33.57% (−48.49 to −14.32%), *P* = 0.008].

Compared to the CMR during 1973–1979, the contribution rates of changing values of CMR caused by demographic factors among the total population during 1980–2019 were over 50%, suggesting that these contribution rates of demographic factors on the changes of CMR were higher than those caused by non-demographic factors. Interestingly, the impact of demographic factors on the change of CMR during 1980–2019 was more evident in males than in females (Supplementary Table 1).

## Discussion

As the largest metropolis and economic center in China, Shanghai stands at the forefront of the national strategic initiatives and responds quickly to the economic reform and health promotion policies. The trend of mortality of liver cancer well reflects the influence of demographic and socioeconomic development on the disease (17). Therefore, Shanghai is one of the most suitable models to clarify the trend in mortality and the impacts of demographic and non-demographic factors on the mortality. Pudong New area, founded in 1993, is the forerunner in China's reform and opening, and it is also the place where urbanization of the rural area occurs most rapidly. The immigration of non-native permanent residents and aging of natives have led to dramatic changes in the population age structure. Thus, Pudong New Area is selected as a study district and is regarded as the microcosm of China's reformation and a good representative of Shanghai (18). In addition, Shanghai has established a cancer registration system since 1973 and become one of the largest cancer registries all over the world. Since 2002, this system has covered 100% of the registered permanent residents and historical record of death registration is intact and accurate, which can provide reliable cancer registration data (13).

The disability-adjusted life-years (DALYs) from liver cancer in China reached 11,530 thousand in 2017, accounting for 53.7% of the global DALYs, so it is essential to understand the mortality and burden caused by liver cancer nationally and regionally (19). In this population-based study, there were significantly decreasing trends of CMR as well as ASMRW of liver cancer from 1973 to 2019 in males, females, and the total population. Consistent with our data, the mortality rates of liver cancer declined in China during 1990–2017 and in Yangpu district of Shanghai during 1974–2014 (20, 21). The mortality rates of liver cancer in people aged over 40 years old continued to decrease possibly attributed to antiviral therapy for the chronic hepatitis B. Since antiviral agent nucleotide/nucleoside analogs were introduced into China in 1998, antiviral therapy has prevented the occurrence of liver cancer and improved the prognosis of patients after hepatectomy (22). Furthermore, the mortality rates of liver cancer among people less than 40 years old declined to some extent due to national HBV vaccination in newborns enforced in mainland China since 1992 (23). Since a large-scale hepatitis B vaccination of newborns was conducted in Taiwan, the mortality rates decreased by up to 70 and 62% in males and females, respectively, during 1996–1999 compared with the 1980–1983 period (24).

Data of mortality by age showed that CMR was at a low level before 30 years old, and it increased rapidly from 45 years old and peaked after 80 years old. For the trends of mortality, increasing trends in the CMR were seen in males from 1973 to 1998 [APC (95% *CI*) = 1.72% (1.20, 2.24%)], and in the total population from 1973 to 1997 [APC (95% *CI*) = 1.57% (1.09, 2.05%)], but the trends began to decrease in 1998 (for males), in 1995 (for females), and in 1997 (for the total population), respectively. Regarding ASMRW, we observed evident decreases after the breakpoint in 1991 (for males), in 1990 (for females), and in 1975 (for the total population), respectively (Table 3). Furthermore, difference decomposition analysis revealed that the contribution caused by demographic factors on the changing value of CMR increased by 45.01% (95% *CI* = 25.87–67.06%) in the total population, by 39.00% (95% *CI* = 22.06–58.29%) in males, and by 46.06% (95% *CI* = 29.06–65.29%) in females (Figure 2). These results suggest that the increase in mortality rate of liver cancer is mainly attributed to demographic factors, especially population aging. Aging is characterized by a progressive decline in efficiency of physiological function with the accumulation of various adverse changes and increased susceptibility to disease. Aging has been demonstrated to be one of the most important risk factors for the occurrence and development of liver cancer (25). Elderly people are vulnerable to hepatocyte aging during chronic liver injury, which is mainly attributed to non-resolving liver inflammation, DNA damage caused by reactive oxygen and nitrogen species, DNA methylation and/or demethylation, and telomere shortening. Moreover, the inactivation of tumor suppressor genes entitles the aging hepatocytes with damaged DNA to escape from cell-cycle arrest and proliferate, and become the candidates for cancer precursor cells (26). Shanghai is the first city entering the aging society in China. In 1982, Pudong New Area entered the aging society, and the proportions of people aged over 60 years old and over 65 years old were 11.37 and 7.72%, respectively. During 1973–2019, the proportion of people aged over 60 years old increased significantly with an APC of 2.90% (95% *CI* = 2.73–3.08%, *P* < 0.001), and the proportion of people aged over 65 years old also showed a significant upward trend (APC = 2.81%, 95% *CI* = 2.66–2.93%, *P* < 0.001) (Supplementary Figure 1). It is estimated that the people aged more than 65 years old in China will reach 322 million by 2050 (12). Although the mortality rates declined in the general population, the rate of people older than 65 years was at a high level. Thus, it can be predicted that death from liver cancer is concentrated in the people aged over 65 years old, and aging should be taken into consideration when designing strategies for the prevention and control of liver cancer.

The concept of YLL due to premature mortality from liver cancer offers a comprehensive and robust measure for monitoring its mortality. YLL rather than standardized rates is better to reflect social and economic impacts on diseases, because it represents an indicator of relative survival considering the age at death (6). YLL gives greater weight to deaths at a younger age than those at an older age. In this study, we found that the age group of 45–59 years rather than the age group of ≥80 years had the highest number of YLL, accounting for 38.3% of the overall YLL due to premature mortality of liver cancer. YLL of liver cancer showed the upward trends in males, females and the total population during 1973–2019, whereas decreasing trends were observed since 1995 and 1996. These changing trends might be attributed to the increasing adoption of surveillance programs, improved therapeutic outcomes obtained in recent decades, surgical advances, and the availability of sorafenib in patients with liver cancer in the advanced stage (27).

The strength of this study is that we provide a comprehensive analysis of mortality and YLL trends covering a long-time span of 1973–2019 (47 years) and a large population size (over 5.0 million), and quantitatively analyze the contribution of demographic and non-demographic factors on the change of mortality rate. As one of the most developed cities of China, Shanghai has experienced dramatic changes in economic and social development. This long-term study provides an example of transition from a developing region to a developed one, and may help in exploring the changes in the spectrum of diseases in the rapid economic and social development of humanity. Nevertheless, some limitations should be noted in interpreting our findings. First, data regarding lifestyle, history of hepatitis and other liver diseases, histological types, and medical care were unavailable, so it was unable to determine the contributions of risk factors to the change of mortality of liver cancer. Second, this study was not individually based, so it was impossible to investigate the association of factors with liver cancer mortality and make causal inferences. Third, as this study covered a 47-year period, the quality of data varied in different periods. The category of liver cancer contains ICD-8, ICD-9, and ICD-10, and potential differential misclassification of the causes of death might be introduced. However, the number of deaths caused by malignant neoplasms remained stable across revisions among different ICD coding systems according to a previous study regarding the comparability of cause of death between ICD-9 and ICD-10 (28).

## Conclusions

In summary, the CMR and ASMRW of liver cancer continually decreased although YLL increased during 1973–2019 in Pudong New Area, the largest district of Shanghai with over five million population. The demographic factors, especially aging, might be responsible for the increase in the mortality of liver cancer. With the health status of aging population getting worse in China and other developing countries, the burden due to the premature mortality of liver cancer is still a challenge for these countries in the future. This registry based study is crucial to provide evidences for authorities to prioritize resources and implement preventive measures to against liver cancer in China and other developing countries.

## Data Availability Statement

The original contributions presented in the study are included in the article/Supplementary Material, further inquiries can be directed to the corresponding author/s.

## Author Contributions

ZL, XL, and YDe drafted the manuscript. XL, YC, and ZL participated in the collection, analysis, and interpretation of data. YDi, YC, LW, JX, and YZ contributed to data collection and suggestion for analysis. XL, LW, and YDe conceived the study, participated in its design and coordination, and critically revised the manuscript. All authors read and approved the final version of the manuscript.

## Funding

This study was funded by the General Program of Health Bureau of Shanghai Pudong New Area (Nos. PW2021A-68 to ZL) Shanghai Health Medical College Faculty Teaching Project (ZPJXKT-21-11 to ZL), the Reserve Academic Leaders Training Program of Pudong New Area Center for Disease Control and Prevention (PDCDC-HBXD2020-05 to XL), the grant from Shanghai Public Health System Construction Three-year Action Plan Outstanding Youth Talent Training Program (GWV-10.2-YQ43 to YC), the Chenzhou Science and Technology Bureau Funds (Nos. ZDYF2017038 to YZ); the Chenzhou Science and Technology Bureau Funds (Nos. ZDYF201816 to HC); the Chenzhou Science and Technology Bureau Funds (Nos. ZDYF2020072 to YZ); the grant from the National Natural Science Foundation of China (Nos. 81803294 to JX).

## Conflict of Interest

The authors declare that the research was conducted in the absence of any commercial or financial relationships that could be construed as a potential conflict of interest.

## Publisher's Note

All claims expressed in this article are solely those of the authors and do not necessarily represent those of their affiliated organizations, or those of the publisher, the editors and the reviewers. Any product that may be evaluated in this article, or claim that may be made by its manufacturer, is not guaranteed or endorsed by the publisher.

## References

[1] Sung H Ferlay J Siegel RL Laversanne M Soerjomataram I Jemal A. Global cancer statistics 2020: GLOBOCAN estimates of incidence and mortality worldwide for 36 cancers in 185 countries. CA Cancer J Clin. (2021) 71:209–49. 10.3322/caac.21660 33538338

[2] Shi JF Cao M Wang Y Bai FZ Lei L Peng J. Is it possible to halve the incidence of liver cancer in China by 2050? Int J Cancer. (2021) 148:1051–65. 10.1002/ijc.33313 32997794

[3] Chen W Zheng R Baade PD Zhang S Zeng H. Cancer statistics in China, 2015. CA Cancer J Clin. (2016) 66:115–32. 10.3322/caac.21338 26808342

[4] Liu Z Mao X Jiang Y Cai N Jin L Zhang T. Changing trends in the disease burden of primary liver cancer caused by specific etiologies in China. Cancer Med. (2019) 8:5787–99. 10.1002/cam4.2477 31385465 PMC6745850

[5] Zhou M Wang H Zeng X Yin P Zhu J Chen W. Mortality, morbidity, and risk factors in China and its provinces, 1990-2017: a systematic analysis for the global burden of disease study 2017. Lancet. (2019) 394:1145–58. 10.1016/S0140-6736(19)30427-1 31248666 PMC6891889

[6] Brustugun OT Møller B Helland A. Years of life lost as a measure of cancer burden on a national level. Br J Cancer. (2014) 111:1014–20. 10.1038/bjc.2014.364 24983370 PMC4150272

[7] Zhang L Li Q Han X Wang S Li P Ding Y. Associations of socioeconomic factors with cause-specific Mortality and burden of cardiovascular diseases: findings from the vital registration in urban Shanghai, China, during 1974-2015. BMC Public Health. (2020) 20:1291. 10.1186/s12889-020-09390-1 32847504 PMC7448450

[8] McGlynn KA Petrick JL El-Serag HB. Epidemiology of hepatocellular carcinoma. Hepatology. (2021) 73:4–13. 10.1002/hep.31288 32319693 PMC7577946

[9] Deng Y Li P Liu W Pu R Yang F Song J. The genetic polymorphism down-regulating HLA-DRB1 enhancer activity facilitates HBV persistence, evolution and hepatocarcinogenesis in the Chinese Han population. J Viral Hepat. (2020) 27:1150–61. 10.1111/jvh.13353 32568442

[10] Sun Y Wang Y Li M Cheng K Zhao X Zheng Y. Long-term trends of liver cancer mortality by gender in urban and rural areas in China: an age-period-cohort analysis. BMJ Open. (2018) 8:e020490. 10.1136/bmjopen-2017-020490 29439081 PMC5829896

[11] Luo Z Lv H Chen Y Xu X Liu K Li X. Years of life lost due to premature death and their trends in people with selected neurological disorders in Shanghai, China, 1995-2018: a population-based study. Front Neurol. (2021) 12:625042. 10.3389/fneur.2021.625042 33746880 PMC7973274

[12] Wang S Du X Han X Yang F Zhao J Li H. Influence of socioeconomic events on cause-specific mortality in urban Shanghai, China, from 1974 to 2015: a population-based longitudinal study. CMAJ. (2018) 190:E1153–61. 10.1503/cmaj.180272 30274992 PMC6167223

[13] Li X Deng Y Tang W Sun Q Chen Y Yang C. Urban-rural disparity in cancer incidence, mortality, and survivals in Shanghai, China, during 2002 and 2015. Front Oncol. (2018) 8:579. 10.3389/fonc.2018.00579 30560091 PMC6287035

[14] Chen H Hao L Yang C Yan B Sun Q Sun L. Understanding the rapid increase in life expectancy in shanghai, China: a population-based retrospective analysis. BMC Public Health. (2018) 18:256. 10.1186/s12889-018-5112-7 29444657 PMC5813363

[15] Luo Z He Y Ma G Deng Y Chen Y Zhou Y. Years of life lost due to premature death and their trends in people with malignant neoplasm of female genital organs in Shanghai, China during 1995-2018: a population based study. BMC Public Health. (2020) 20:1489. 10.1186/s12889-020-09593-6 33004024 PMC7528500

[16] Cheng X Yang Y Schwebel DC Liu Z Li L Cheng P. Population ageing and mortality during 1990-2017: a global decomposition analysis. PLoS Med. (2020) 17:e1003138. 10.1371/journal.pmed.1003138 32511229 PMC7279585

[17] Are C Meyer B Stack A Ahmad H Smith L Qian B. Global trends in the burden of liver cancer. J Surg Oncol. (2017) 115:591–602. 10.1002/jso.24518 28345140

[18] Li X Qian M Zhao G Yang C Bao P Chen Y. The performance of a community-based colorectal cancer screening program: evidence from Shanghai Pudong New Area, China. Prev Med. (2019) 118:243–50. 10.1016/j.ypmed.2018.11.002 30412744

[19] GBD 2017 Causes of Death Collaborators. Global, regional, and national age-sex-specific mortality for 282 causes of death in 195 countries and territories, 1980-2017: a systematic analysis for the Global Burden of Disease Study 2017. Lancet. (2018) 392:1736–88. 10.1016/S0140-6736(18)32203-7 30496103 PMC6227606

[20] Wang F Mubarik S Zhang Y Wang L Wang Y Yu C. Long-term trends of liver cancer incidence and mortality in China 1990-2017: a join point and age-period-cohort analysis. Int J Environ Res Public Health. (2019) 16:2878. 10.3390/ijerph16162878 31408961 PMC6719938

[21] Li M Wang S Han X Liu W Song J Zhang H. Cancer mortality trends in an industrial district of Shanghai, China, from 1974 to 2014, and projections to 2029. Oncotarget. (2017) 8:92470–82. 10.18632/oncotarget.21419 29190931 PMC5696197

[22] Yin J Wang J Pu R Xin H Li Z Han X. Hepatitis B virus combo mutations improve the prediction and active prophylaxis of hepatocellular carcinoma: a clinic-based cohort study. Cancer Prev Res (Phila). (2015) 8:978–88. 10.1158/1940-6207.CAPR-15-0160 26290395

[23] Tang X Allain JP Wang H Rong X Chen J Huang K. Incidence of hepatitis B virus infection in young Chinese blood donors born after mandatory implementation of neonatal hepatitis B vaccination nationwide. J Viral Hepat. (2018) 25:1008–16. 10.1111/jvh.12901 29624818

[24] Lee CL Hsieh KS Ko YC. Trends in the incidence of hepatocellular carcinoma in boys and girls in Taiwan after large-scale hepatitis B vaccination. Cancer Epidemiol Biomarkers Prev. (2003) 12:57–9. 12540504

[25] Fujiwara N Tateishi R Kondo M Minami T Mikami S Sato M. Cause-specific mortality associated with aging in patients with hepatocellular carcinoma undergoing percutaneous radiofrequency ablation. Eur J Gastroenterol Hepatol. (2014) 26:1039–46. 10.1097/MEG.0000000000000161 25051219

[26] Nakajima T Nakashima T Yamaoka J Shibuya A Konishi E Okada Y. Greater age and hepatocellular aging are independent risk factors for hepatocellular carcinoma arising from non-B non-C non-alcoholic chronic liver disease. Pathol Int. (2011) 61:572–6. 10.1111/j.1440-1827.2011.02743.x 21951665

[27] Cucchetti A Trevisani F Bucci L Ravaioli M Farinati F Giannini EG. Years of life that could be saved from prevention of hepatocellular carcinoma. Aliment Pharmacol Ther. (2016) 43:814–24. 10.1111/apt.13554 26864152

[28] Cen X Wang D Sun W Cao L Zhang Z Wang B. The trends of mortality and years of life lost of cancers in urban and rural areas in China, 1990-2017. Cancer Med. (2020) 9:1562–71. 10.1002/cam4.2765 31873982 PMC7013076

