# Supplementary material for: Influence of Demographic Factors on Long-Term Trends of Premature Mortality and Burden Due to Liver Cancer: Findings From a Population-Based Study in Shanghai, China, 1973–2019
Source: Front Public Health. 2022 Feb 15;10:808917. doi: 10.3389/fpubh.2022.808917 (PMC8885583; doi:10.3389/fpubh.2022.808917)
Supplement: Supplementary file 1 [file Table_1.DOCX]

***Supplementary Materials***

**Influence of demographic factors on long-term trends of** **premature mortality and burden due to liver cancer: findings from a population-based longitudinal study in Shanghai, China, 1973-2019**

***Zheng Luo ^†^, Yongbin Zou ^†^, Jiaxin Xie ^†^, Hui Cao, Yichen Chen, Yibo Ding, Xiaopan Li ^*^, Yang Deng ^*^ and Lile Wu ^*^***

***^*^Correspondence:*** *Xiaopan Li: xiaopanli0224@126.com.*

*Yang Dengl: dengyang3417@126.com.*

*Lile Wu: wbm_bigcat@hotmail.com.*


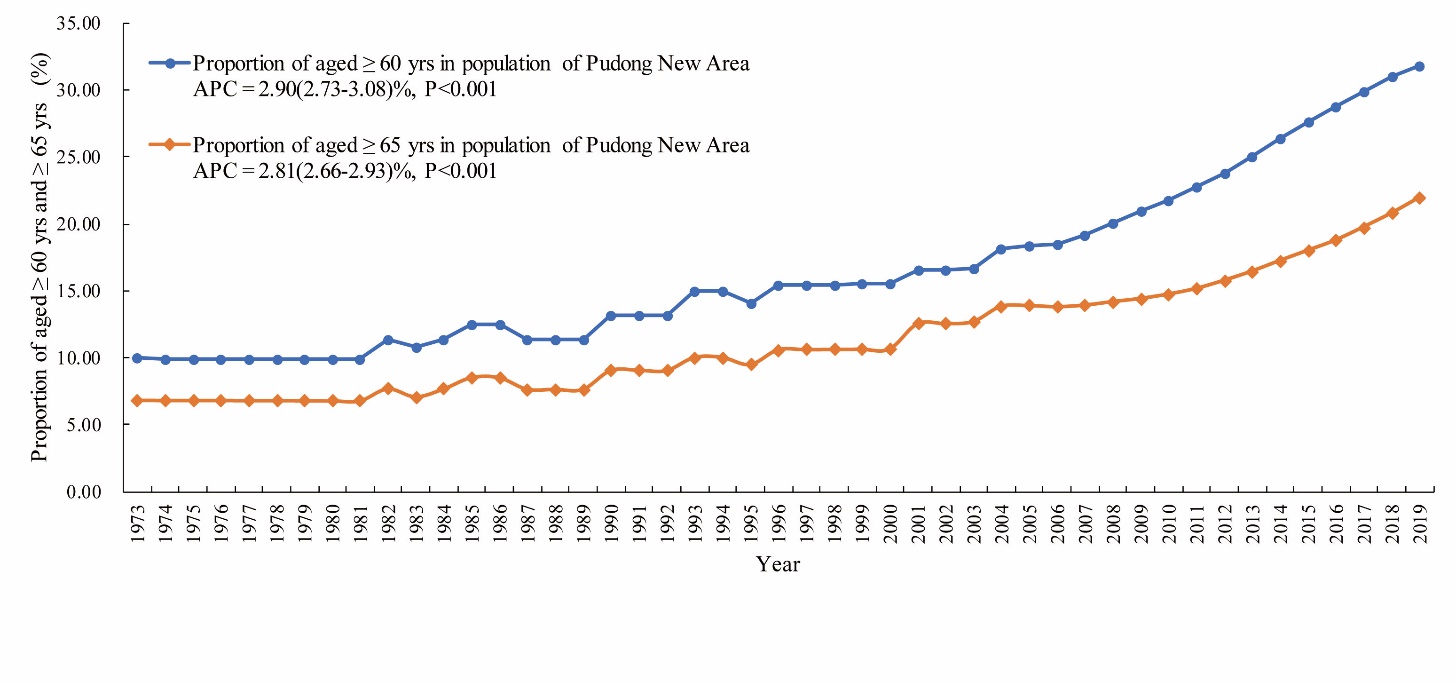


**Supplementary Figure 1** | The proportion of people aged over 60 years and over 65 years in Pudong New Area, Shanghai, China, 1973-2019.

**Supplementary Table 1** | The changing rates caused by demographic and non-demographic factors and their contribution rates during the period from 1980 to 2019 compared with the CMR of liver cancer during 1973-1979 in genders

| **Characteristic** | **CMR of based period (/10^5^)** | **CMR of the other period (/10^5^)** | **D-value of CMR (/10^5^)** | **Impact of demographic factors** | |  | **Impact of non-demographic factors** | |
| --- | --- | --- | --- | --- | --- | --- | --- | --- |
|  |  |  |  | **Changing value (/10^5^)** | **Contribution rate (%)** |  | **Changing value (/10^5^)** | **Contribution rate (%)** |
| Male |  |  |  |  |  |  |  |  |
| 1980-1984 vs. 1973-1979 | 30.57 | 33.89 | 3.32 | 3.31 | 99.89 |  | 0.01 | 0.11 |
| 1985-1989 vs. 1973-1979 | 30.57 | 39.03 | 8.46 | 7.41 | 87.63 |  | 1.05 | 12.37 |
| 1990-1994 vs. 1973-1979 | 30.57 | 38.76 | 8.19 | 15.66 | 67.71 |  | -7.47 | 32.29 |
| 1995-1999 vs. 1973-1979 | 30.57 | 44.46 | 13.88 | 20.29 | 76.00 |  | -6.41 | 24.00 |
| 2000-2004 vs. 1973-1979 | 30.57 | 42.18 | 11.60 | 26.19 | 64.23 |  | -14.59 | 35.77 |
| 2005-2009 vs. 1973-1979 | 30.57 | 38.04 | 7.47 | 32.45 | 56.50 |  | -24.98 | 43.50 |
| 2010-2014 vs. 1973-1979 | 30.57 | 35.81 | 5.24 | 36.77 | 53.84 |  | -31.53 | 46.16 |
| 2015-2019 vs. 1973-1979 | 30.57 | 30.97 | 0.40 | 38.81 | 50.26 |  | -38.41 | 49.74 |
| APPC (95% *CI*) | - | - | - | 39.00 (22.06, 58.29)^**^ | - |  | -38.20 (-55.40 ,-14.37)^*^ | - |
| Female |  |  |  |  |  |  |  |  |
| 1980-1984 vs. 1973-1979 | 17.48 | 16.52 | -0.97 | 1.21 | 35.75 |  | -2.18 | 64.25 |
| 1985-1989 vs. 1973-1979 | 17.48 | 19.04 | 1.56 | 2.77 | 69.58 |  | -1.21 | 30.42 |
| 1990-1994 vs. 1973-1979 | 17.48 | 18.21 | 0.72 | 6.39 | 53.00 |  | -5.66 | 47.00 |
| 1995-1999 vs. 1973-1979 | 17.48 | 19.65 | 2.16 | 7.97 | 57.85 |  | -5.81 | 42.15 |
| 2000-2004 vs. 1973-1979 | 17.48 | 17.01 | -0.47 | 10.89 | 48.95 |  | -11.36 | 51.05 |
| 2005-2009 vs. 1973-1979 | 17.48 | 15.96 | -1.52 | 14.88 | 47.57 |  | -16.40 | 52.43 |
| 2010-2014 vs. 1973-1979 | 17.48 | 14.47 | -3.01 | 17.56 | 46.05 |  | -20.57 | 53.95 |
| 2015-2019 vs. 1973-1979 | 17.48 | 13.75 | -3.73 | 20.30 | 45.79 |  | -24.03 | 54.21 |
| APPC (95% *CI*) | - | - | - | 46.06 (29.06, 65.29)^**^ | - |  | -32.74 (-45.70, -16.68)^*^ | - |
| Total |  |  |  |  |  |  |  |  |
| 1980-1984 vs. 1973-1979 | 23.43 | 24.61 | 1.18 | 1.60 | 79.31 |  | -0.42 | 20.69 |
| 1985-1989 vs. 1973-1979 | 23.43 | 28.52 | 5.09 | 3.96 | 77.73 |  | 1.13 | 22.27 |
| 1990-1994 vs. 1973-1979 | 23.43 | 28.29 | 4.86 | 9.14 | 68.13 |  | -4.27 | 31.87 |
| 1995-1999 vs. 1973-1979 | 23.43 | 31.98 | 8.56 | 11.82 | 78.35 |  | -3.27 | 21.65 |
| 2000-2004 vs. 1973-1979 | 23.43 | 29.59 | 6.16 | 15.88 | 62.04 |  | -9.72 | 37.96 |
| 2005-2009 vs. 1973-1979 | 23.43 | 27.02 | 3.59 | 20.56 | 54.78 |  | -16.97 | 45.22 |
| 2010-2014 vs. 1973-1979 | 23.43 | 25.11 | 1.69 | 23.72 | 51.84 |  | -22.03 | 48.16 |
| 2015-2019 vs. 1973-1979 | 23.43 | 22.33 | -1.09 | 25.99 | 48.97 |  | -27.08 | 51.03 |
| APPC (95% *CI*) | - | - | - | 45.01 (25.87, 67.06)^**^ | - |  | -33.57 (-48.49, -14.32)^*^ | - |

APPC, average period percentage change; CMR, crude mortality rate; D-value, difference value.
